# Perceptions and Adaptation Strategies in Fundamental Movement Skills Among Children With Physical Disabilities

**DOI:** 10.1177/00315125251412691

**Published:** 2025-12-24

**Authors:** Margaux Hebinck, Krista L. Best, Maxime T. Robert

**Affiliations:** 1 School of Rehabilitation Sciences, 4440 Laval University, Quebec, QC, Canada; 2 Interdisciplinary Research Centre on Rehabilitation and Social Integration, Quebec, QC, Canada

**Keywords:** motor development, child participation, movement assessment, inclusive practices, individualized intervention

## Abstract

Fundamental movement skills (FMS) are foundational to children’s motor development and long-term participation in physical activity. However, standardized assessments often fail to capture the diverse abilities and lived experiences of children with physical disabilities. This qualitative study explored how these children perceive and perform FMS. Seventeen participants ages 3 to 10 took part in two sessions: one to assess their FMS using the Test of Gross Motor Development, the 3rd edition, and another to discuss their understanding of specific skills through an unstructured interview. Thematic analysis of verbal and non-verbal data revealed three key themes. First, children’s knowledge, motivation, and perceived competence varied widely, influenced by age, fatigue and task familiarity. Younger children tended to show strong confidence, while older children were more self-critical. Second, children used a variety of motor, material, environmental, and contextual adaptation strategies to support FMS execution, often co-constructed with the research team. These adaptations reflected individual needs more than task-specific demands. Third, children focused more on outcomes than on movement quality and had difficulty assessing their own performance. Visual feedback and playful contexts supported engagement and self-awareness. These results highlight the need for inclusive, personalized approaches that integrate both process- and product-oriented perspectives and actively involve children in shaping their learning experience. Future research should investigate how such strategies can be integrated into children’s everyday environments.

## Introduction

Fundamental movement skills (FMS) refer to basic movement patterns, including locomotion skills (e.g., running, jumping) and object control skills (e.g., throwing, catching) (Goodway et al., 2019; Metcalfe & Clark, 2002). These skills are foundational to children’s motor development and support participation in a wide range of physical activities (Goodway et al., 2019; Stodden et al., 2008). According to the motor development model proposed by Goodway and colleagues, FMS typically emerge during the fundamental movement phase (ages 2-7) and continue to evolve into more specialized and context-specific skills during the specialized movement phase, which begins after age 7 (Goodway et al., 2019). Early acquisition of FMS is strongly associated with long-term physical activity engagement, improved physical health and enhances psychological and social well-being (Cairney et al., 2019; Lubans et al., 2010; Robinson et al., 2015).

FMS are commonly assessed using two main approaches: process-oriented measures, which examine the quality of movement execution, and product-oriented measures, which focus on performance outcomes (e.g., jumping distance or throwing accuracy) (Burton & Miller, 1998; Logan et al., 2018; Payne & Isaacs, 2025). Standardized tools, such as the Test of Gross Motor Development (TGMD), are based on data from typically developing children (Ulrich, 2013). While these tools are valuable for identifying general motor delays (Bonney & Smits-Engelsman, 2019; Cools et al., 2009), they may not fully capture the nuanced and often non-linear developmental trajectories of children with physical disabilities, whose motor progress may be shaped by distinct sensory, motor or perceptual factors (Adolph & Hoch, 2019; Klavina et al., 2017; Rosenbaum & Gorter, 2012). A recent scoping review confirmed that children with physical disabilities consistently show lower FMS proficiency than their typically developing peers (Hebinck et al., 2025). These differences are compounded by assessments that overlook the use of assistive devices, environmental barriers, and creative compensatory strategies. As a result, children are often evaluated using criteria that may not fully capture their abilities. Such conventional assessment risks reinforcing ableist assumptions about standardized or stereotypical forms of movement, which may not reflect the realities of children with physical disabilities (Lyons, 2013; Pushkarenko et al., 2023).

To address these limitations, rehabilitation approaches are increasingly shifting toward holistic, person-centered models (Gibson et al., 2009; Imms et al., 2017; Rosenbaum & Gorter, 2012). These models go beyond simply correcting impairments to recognize and build on individuals’ strengths, preferences, and life contexts (Imms et al., 2017; World Health Organization, 2001). Interventions aim not only to improve motor and functional skills but also to remove barriers to social participation, promote autonomy, motivation and a sense of belonging, and support the child’s overall engagement in meaningful environments (Adair et al., 2018; Imms et al., 2016; King et al., 2003; Rosenbaum & Gorter, 2012). Within this perspective, the participation-centered approach has emerged as a central framework in practice, guiding interventions aimed at fostering meaningful engagement of children in their daily environments (Imms et al., 2016, 2017; King et al., 2003). This approach emphasizes interventions that are contextualized, motivating and co-constructed with the child and family, anchoring therapeutic goals in inclusive, meaningful and sustainable activities (Imms et al., 2016; Palisano et al., 2012; Rosenbaum & Gorter, 2012). Closely aligned with this vision, the strengths-based approach although not yet fully theorized. It aims to reinforce what the child is already capable of, using their strengths as a pathway to participation rather than focusing solely on skill acquisition (Rosenbaum & Gorter, 2012).

Within this broader context, precision rehabilitation has emerged as a promising approach. Precision rehabilitation seeks to tailor interventions to each child’s unique motor, sensory, cognitive, emotional and environmental profile (French et al., 2022). Grounded in a deeply individualized logic, this approach aims to optimize outcomes by responding to the child’s real needs and specific responses (Imms et al., 2017; Rosenbaum & Gorter, 2012). Precision rehabilitation shares key principles with participation- and strengths-based approaches. Motor competence could, therefore, be seen not as conformity to norms, but as the child’s ability to perform a task meaningfully, independently and confidently (Hulteen et al., 2018; Rudd et al., 2020). What might be labelled as an “error” in a standardized assessment can instead reflect a functional, intentional and effective adaptation to the child’s body or environment (Chow et al., 2021; Rudd et al., 2020). This perspective is consistent with Newell’s constraints-led framework, where observed “errors” may instead reflect functional adaptations to organismic, task, and environmental constraints (Balagué et al., 2019). Similarly, ecological dynamics perspectives highlight that learners navigate performance landscapes by developing functional movement solutions that may not conform to standardized norms but are nevertheless effective (Rudd et al., 2021; Woods et al., 2020). Precision rehabilitation thus encourages practitioners to support and reinforce these individualized strategies as authentic expressions of the child’s potential.

A key step toward more personalized intervention is integrating the perspectives of children themselves (Imms et al., 2017; Shields & Synnot, 2016). While most existing literature emphasizes performance outcomes, to the best of our knowledge, no studies have directly explored how children with physical disabilities interpret motor tasks, understand their own strategies, or identify helpful forms of support. These key understandings are crucial for designing interventions that align with individuals lived experiences. Prior research has demonstrated that children want to be heard, involved in decisions, and supported in ways that reflect their individual needs and interests (Clark, 2005; Lundy, 2007). They value fun, peer relationships, and individualized support (Lauruschkus et al., 2015). Participation facilitators and barriers are highly personal; there is no one-size-fits-all approach (Shields & Synnot, 2016). Although few studies have directly explored how children’s voices can shape the development of FMS, a study demonstrated that incorporating student voices into FMS programs can enhance autonomy, motivation, and engagement, thereby supporting the idea that listening to what matters to children allows practitioners to design more meaningful and effective tasks (O’Sullivan et al., 2020). Recognizing children as active participants in their motor learning resonates with their results, which highlight the potential for greater enjoyment and long-term progress. Listening to what matters to children shifts the focus from normative expectations to inclusive, individualized, and experience-based approaches (Imms et al., 2016; O’Sullivan et al., 2020).

In this context, the aim of this study was to explore how children with physical disabilities perceive and perform FMS. Specifically, we investigated how children understood their FMS and identified various spontaneous and guided adaptation strategies that supported their performance.

## Methods

### Design

A descriptive qualitative design was conducted using video recordings to capture both verbal and non-verbal data (Colorafi & Evans, 2016; Sandelowski, 2000, 2010). This study was grounded in a constructivist paradigm, which acknowledges the existence of multiple subjective realities and values the co-construction of meaning between participants and researchers. This approach was chosen to explore the individual perceptions and experiences of children with physical disabilities in depth. Ethical approval was obtained from the *Centre intégré universitaire de santé et de services sociaux de la Capitale-Nationale (CIUSSS-CN)* Research Ethics Committee (#2021-2320). Written informed consent and assent for children were obtained from all participants and their legal guardians before participation. To ensure rigour, the results are reported using the Consolidated criteria for Reporting Qualitative research checklist (Tong et al., 2007) (Supplemental Material 1).

### Participants

To be included in the study, participants were children with a diagnosis of physical disability (e.g., developmental coordination disorder, cerebral palsy, or limb amputation) and aged between three and ten years and eleven months, as defined by the age range specified in the TGMD-3 assessment tool (Ulrich, 2013). To ensure comprehension and meaningful participation, children were required to understand spoken French and be able to communicate in French. Children were excluded if they had a primary diagnosis of cognitive or sensory disability, as these conditions could significantly affect their ability to understand instructions or perform motor tasks independently. Children were also excluded if they had undergone upper or lower limb surgery within the six months prior the data collection, due to potential impacts on motor function and recovery.

Participants were recruited using convenience sampling (Golzar et al., 2022). A recruitment flyer was sent by email to all families of children either currently receiving therapy or on the waiting list for programs at the pediatric site of the *CIUSSS-CN*. In addition, the flyer was shared via social media platforms affiliated with the Interdisciplinary Research Center for Rehabilitation and Social Integration, as well as the Clinical Neurodevelopment Laboratory. Interested families were invited to contact the research team directly or provide consent to be contacted. The first author then followed up with legal guardians by email or phone to provide detailed information about the study and confirm eligibility.

### Procedure

#### Session 1. Motor skills assessment

Sociodemographic data, including age, sex, diagnosis, and the use of mobility aids, were collected for all participants. These questionnaires were completed using a computer by the legal guardians, with input from the children, prior to the first session. Each child participated in an initial session during which their FMS were assessed using the TGMD-3, a validated and reliable motor assessment designed for children aged 3 to 10 years and 11 months (Ulrich, 2013). The TGMD-3 evaluates 13 motor skills divided into two subscales: locomotor skills (e.g., running, hopping, galloping) and object control skills (e.g., catching, kicking, throwing). Each skill was assessed across two trials, with performance rated according to standardized criteria. For each trial, every criterion was scored as 1 (successful) or 0 (unsuccessful), giving each child two attempts per skill. All sessions were video recorded using a tablet device by the first author, ensuring consistent framing and perspective across participants. Scoring was conducted post-assessment sessions using video recordings, allowing for detailed analysis at different playback speeds, including slow motion, to ensure precisely scoring of complex performance criteria (e.g., hopping, horizontal jumping) (Rintala et al., 2017). The initial assessment and all subsequent scoring were carried out by the first author, who was trained in the standardized administration and scoring procedures of the TGMD-3 to ensure consistency with validated methods. The session took place in a gymnasium at the pediatric site of the *CIUSSS-CN.*

#### Session 2. Discussion about FMS

Following the initial assessment, children participated in a second session, lasting between 60 and 90 minutes, focused on discussing and exploring the FMS for which they had received scores at or below the average for each skill (typically between three and five points per skill; Ulrich, 2013). This session was intended to gain a deeper understanding of how children perceive these skills, the challenges they face, and the spontaneous or guided strategies they use or consider to improve their performance. While the overall structure of the session was consistent across participants (Figure 1), the discussions were designed to be open-ended and child-led, reflecting the format of an unstructured interview (Chauhan, 2022). A flexible guide was used, but the first author had substantial freedom to follow the child’s pace, interests, and responses, allowing for organic exploration of the phenomenon (Chauhan, 2022). Depending on the child’s age, attention span and fatigue level, four to five skills were selected for in-depth exploration. These factors were assessed informally through behavioural cues (e.g., restlessness, verbal fatigue, loss of engagement), rather than via formal testing. All sessions took place in the same environment as the initial assessment and were facilitated by the first author. Parents were present during the session but did not contribute to the content of the discussions. Their role was limited to supporting their child, when necessary (e.g., providing reassurance, clarifying instruction, or encouraging participation). Each session was video recorded using a digital tablet.

**Figure 1.**
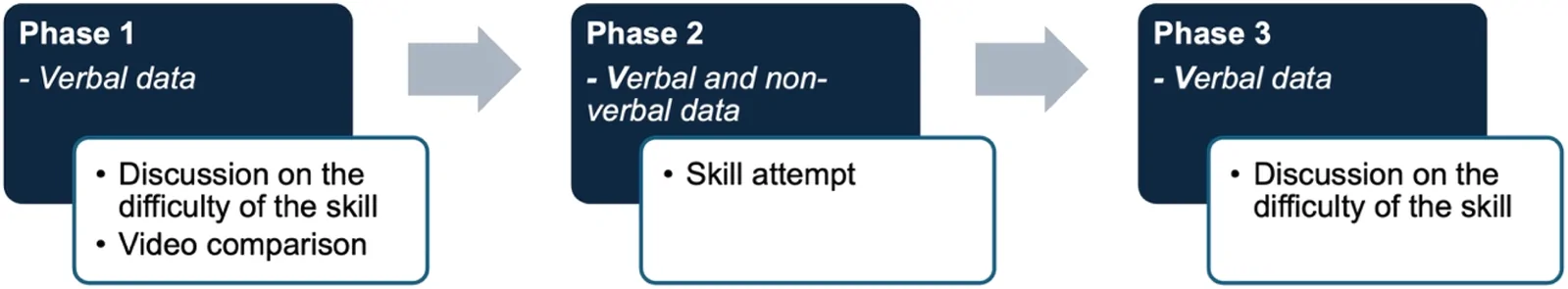
The process of data collection in session 2

Data collection integrated both spontaneous conversations and movement trials and was organized into three flexible phases. ***Phase 1.*** The session began with an exploratory discussion where children were invited to reflect on the perceived difficulty of a given skill based on its name *(i.e., « If I say [name of the skill], does it seem easy or difficult to you? Would you be able to explain why? »)*. To support this reflection, children attempted the skill and were encouraged to share any feeling, associations or past experiences related to it. Following this, participants watched two videos: the first showed a model performing the skill according to TGMD-3 performance criteria, illustrating the expected components of the movement; the second showed the participant’s own performance during the initial assessment, allowing for comparison and reflection. Children were asked to reflect on the differences between the two recordings *(i.e., « Do you see any differences between these two videos? »)*, and on their ability to perform the skill as shown in the demonstration *(i.e., « Do you feel able to perform it as in the demonstration video? »)*. ***Phase 2.*** Children were first invited to think about and try their own spontaneous adaptive strategies. Only after they had the opportunity to generate and test these self-initiated adaptations, or when a child did not propose any strategy, were additional options introduced. These researcher-provided strategies, drawn from the scientific literature (Supplemental Material 2; Colvin et al., 2023; Department of Education Western Australia, 2013), were presented consistently across participants and only after the child had attempted the task independently. Throughout this phase, participants were encouraged to verbalize their preferences, justify their strategy choices, and discuss how these adjustments felt or helped in relation to their abilities and needs. This phase remained highly adaptable depending on each child’s responses and level of engagement. ***Phase 3.*** Children were asked to reassess the perceived difficulty of the skill after trying various adaptations *(i.e., « Now that we have practiced the skill using new strategies, does [name of the skill] seem easy or difficult for you? »)*. This discussion aimed to capture how children’s perceptions evolved and how they interpreted usefulness or different strategies in improving their performance.

### Data Analysis

Descriptive statistics (mean, standard deviation, and frequency) were calculated for both sociodemographic data and FMS raw scores. The raw scores for each skill were calculated using the TGMD-3 scoring system. Based on Gallahue’s motor development model, participants were categorized into three age groups: 3–5 years (Fundamental movement phase – Emerging elementary stage), 6-7 years (Fundamental movement phase – Proficient stage), and 8–10 years (Specialized movement phase – Transitional stage) (Goodway et al., 2019).

The video data collected during the sessions were analyzed using a reflexive thematic analysis, with an inductive approach, following the six-step process described by Braun and Clarke (2006): (1) familiarization with the data through repeated viewing and transcription of key segments; (2) initial code generation from the children’s verbal and non-verbal expressions, as well as researcher observations; (3) systematic organization of codes into potential themes; (4) review and refinement of themes based on relevance and recurrence across participants; (5) definition and naming of final themes; and (6) report writing with illustrative examples (Braun & Clarke, 2006). To further clarify how non-verbal behaviours were processed, an example of a meaningful observation included in the thematic analysis is provided. During several tasks, some children remained still for a few seconds before performing the movement or responding verbally. These short sequences were identified as meaningful information, as they reflected hesitation or decision-making prior to action. They were coded as “nonverbal reaction to the task”. This coding was then reviewed and refined through team discussions by comparing it with similar behaviours observed across participants and ensuring its relevance to the overall analysis.

The analysis was based on three complementary data sources: children’s verbal responses, their non-verbal behaviours and researcher observations documented in a reflexive journal. These data types were triangulated to enrich interpretation and capture multiple layers of meaning, allowing for a more comprehensive understanding of children’s experiences. The first author, who conducted all the sessions, also led the coding process. She adopted a reflexive stance throughout the analysis, using a journal to record impressions, questions and analytical decisions. An initial codebook was developed based on initial observations and iteratively refined as new patterns emerged. All video recordings were manually coded by the first author (M.H) using *Microsoft Excel* (version 16.98). The coding, categories and themes phases were regularly discussed with the research team. As the sessions were conducted in French, all verbatim excerpts were translated into English and verified by two bilingual team members (K.L.B and M.T.R).

### Researcher Bias and Trustworthiness

Data collection and analysis were conducted by the first author, a female doctoral student in rehabilitation sciences with a background in adapted physical activity, health and motor development. Although she had no prior relationship with the children or their families, her expertise in the field may have influenced the interpretation of certain interactions. Furthermore, all members of the research team were trained in kinesiology and shared professional expertise in physical activity. While this disciplinary alignment provided valuable insight into motor behaviours, it also required heightened awareness of potential confirmation biases, particularly regarding implicit expectations about competence or participation. Aware of these potential influences, a reflective stance was maintained throughout the process. The first author kept a detailed reflexive journal, documenting impressions, analytical decisions, questions and emerging interpretations. This ongoing reflexive process supports transparency and accountability in analytical reasoning.

Several strategies consistent with Lincoln and Guba’s (1985) criteria were implemented to enhance the trustworthiness of the analysis (Lincoln & Guba, 1985). Credibility was supported through triangulation of data sources (verbal and non-verbal), deep immersion in the video material and regular, in-depth discussions with the research teams during both data collection and analysis. These discussions were not confined to isolated meetings but unfolded as an iterative process involving repeated cycles of theme generation and interpretation. Divergent interpretations were actively debated and re-examined, considering raw data, ensuring that the resulting themes were co-constructed and empirically grounded. This collaborative and reflexive approach aligns with Braun and Clarke’s (2006) emphasis on transparency and researcher subjectivity, rather than seeking intercoder reliability (Braun & Clarke, 2006). Confirmability was reinforced through the reflexive journal, which served as an audit trail of the analytical process. Transferability was supported by providing detailed descriptions of the study context, participant profiles, and interactional settings, enabling readers to assess the relevance of the findings to other populations or contexts.

## Results

A total of 19 children participated in the study. During the initial assessment session, two participants demonstrated significant difficulties with verbal and non-verbal comprehension, as well as verbal expression. To prevent potential frustration or a sense of failure, these participants were not invited to complete the second session. Consequently, the full assessment protocol was completed and analyzed for 17 children, representing 89% of the recruited sample.

Among these 17 children, seven were girls and ten were boys, with a mean age of 6.8 years (±2.7). Age distribution was as follows: five children were between 3–5 years, four were 6–7 years and eight were 8–10 years. The participants had a variety of diagnoses: thirteen children had impairments involving both upper and lower limbs (e.g., developmental coordination disorder (DCD)), three had limitations restricted to the upper limb (e.g., congenital hypoplasia), and one primarily experienced difficulty with the lower limb (e.g., amputation). Three children used mobility aids, including orthoses, a leg prothesis, and a walker.

Figure 2 presents the fundamental movement skills that were discussed with the children during the second session. The skills discussed with each child and their personal characteristics (age group, diagnosis, use of a mobility aid) are presented in Table 1.

**Figure 2.**
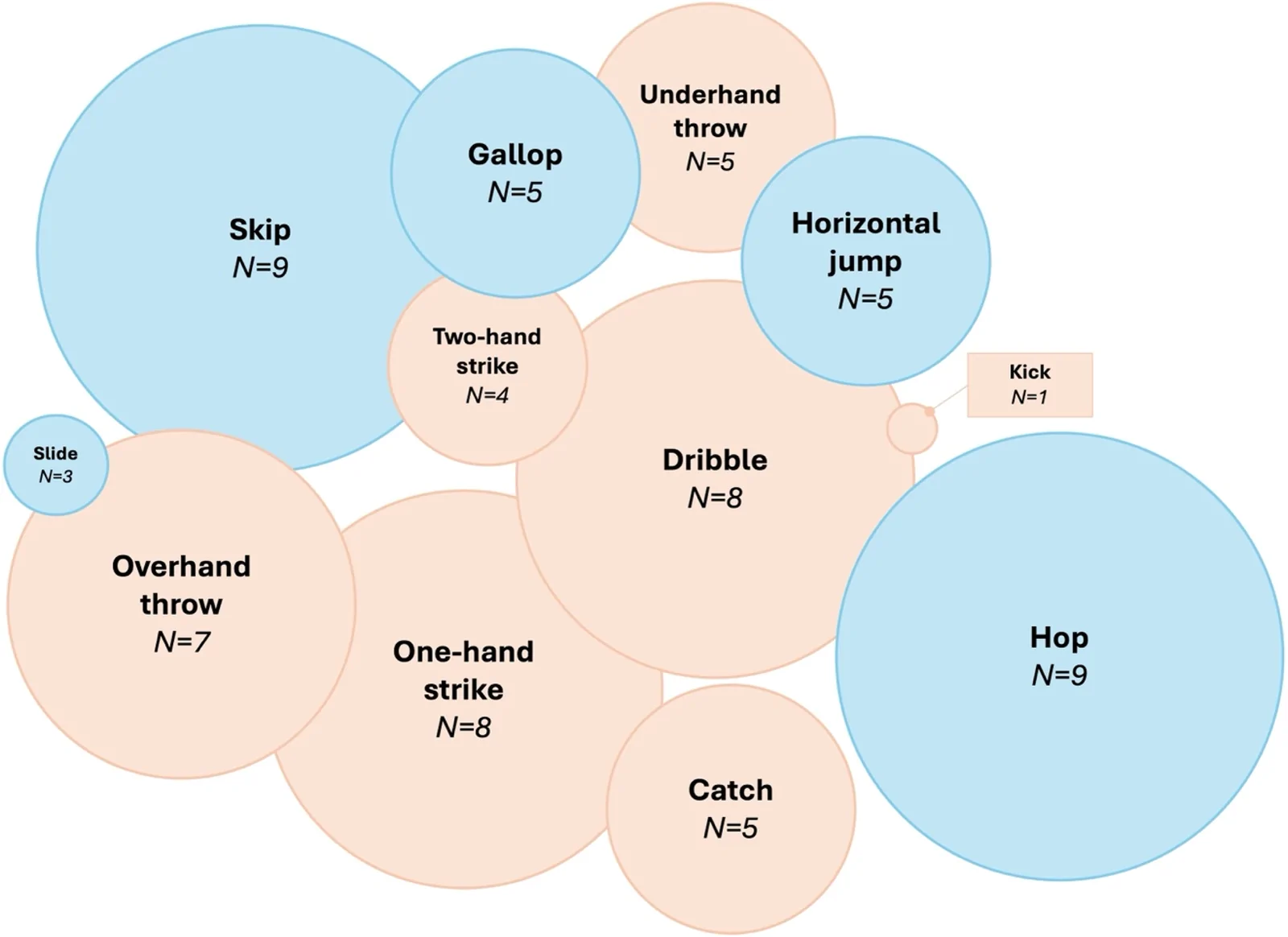
Frequency of discussion of fundamental movement skills by type of skills (locomotor or object control). The size of each circle is proportional to the number of children (N) who discussed that particular skill. The skills are grouped into two categories: locomotor skills (blue circles) and object control skills (orange circles)

**Table 1.** Overview of Participants: Age Group, Diagnosis, Mobility Aids, and Discussed Motor Skills

Participant codes	Age group	Diagnostic	Mobility aids	Locomotion skills	Object control skills
FMS_005	3–5 years	Motor delay and hyperlaxity	-	• Gallop	• Two-hand strike
				• Hop	• Dribble
FMS_011	3–5 years	Agenesis with adactyly *(left hand)*	-	• Hop	• Catch
				• Slide	• Underhand throw
FMS_013	3–5 years	Hypotonia and growth delay	Ankle orthoses	• Hop	• Dribble
				• Horizontal jump	• Overhand throw
FMS_014	3–5 years	Congenital hypoplasia *(left hand)*	-	• Gallop	• One-hand strike
					• Dribble
					• Catch
FMS_017	3–5 years	Spina bifida	-	• Hop	• Dribble
				• Skip	
FMS_003	6–7 years	Motor development delay	-	• Skip	• Dribble
				• Horizontal jump	• Underhand throw
FMS_004	6–7 years	Motor learning difficulties with elements of development coordination disorder	-	• Hop	• Catch
				• Skip	• Overhand throw
				• Horizontal jump	
FMS_008	6–7 years	Non-verbal learning disorder syndrome and development coordination disorder	-	• Hop	• One-hand strike
				• Skip	• Overhand throw
					• Underhand throw
FMS_019	6–7 years	Cerebral palsy	Walker	• Skip	• Overhand throw
				• Horizontal jump	• Catch
				• Slide	
FMS_001	8–10 years	Development coordination disorder	-	• Gallop	• One-hand strike
				• Hop	• Dribble
					• Overhand throw
FMS_002	8–10 years	Development coordination disorder, hyperlaxity and hypotonia	-	• Gallop	• Two-hand strike
				• Skip	• Catch
FMS_006	8–10 years	Fine motor and balance disorder	-	• Hop	• One-hand strike
					• Two-hand strike
					• Overhand throw
FMS_009	8–10 years	Development coordination disorder	-	-	• Two-hand strike
					• One-hand strike
					• Dribble
FMS_010	8–10 years	Elbow malformation	-	• Gallop	• One-hand strike
				• Skip	• Underhand throw
FMS_012	8–10 years	Development coordination disorder	-	• Horizontal jump	• Two-hand strike
					• Dribble
					• Overhand throw
FMS_016	8–10 years	Transtibial amputation *(left leg)*	Leg prothesis	• Skip	• Kick
				• Slide	
FMS_018	8–10 years	Ehlers-Danlos syndrome and Hyperlaxity	-	• Hop	• One-hand strike
				• Skip	• Underhand throw

The analysis revealed three main themes. The first highlights the variability in knowledge, motivation, and perceived competence in fundamental movement skills. The second identifies adaptation strategies that influence the execution of fundamental movement skills. This theme is divided into four distinct subthemes: motor strategies, material strategies, environmental strategies and contextual strategies. Finally, the third theme underscores the diversity of perceptions regarding skill execution.

### Theme 1: Variability in Knowledge, Motivation, and Perceived Competence in Fundamental Movement Skills

The findings revealed a varying level of knowledge of FMS among children. Object control skills, as well as common locomotor actions (i.e., running and jumping) were generally well recognized. In these cases, just hearing the name of the skill was sufficient for the child to perform it spontaneously, without the need for clarification or demonstration. Some children were so familiar with these skills that they immediately positioned themselves to execute the movement, even before asking them. In contrast, other skills, such as galloping, skipping or sliding, were frequently unfamiliar or poorly understood, regardless of age. When the name alone was not sufficient to trigger recognition, children were unable to perform the corresponding skill and required demonstration and video modeling to understand what was expected. For example, when shown a demonstration of “sliding”, some children were able to recognize and understand the skill more easily. Children’s recognition of certain motor skills seemed to improve when these are presented within familiar physical activity contexts. For example, striking a stationary ball was more readily understood when linked to baseball, while striking a moving ball naturally evoked tennis. Although thematic analysis does not rely on quantification, a descriptive table was included (Supplemental Material 3) to provide additional contextual information about participants’ spontaneous recognition of the skills by name.

From a motivational standpoint, children generally displayed enthusiasm for practising skills and trying new activities. However, motivation tended to decrease over the course of the session, particularly due to fatigue, which was observed across all age groups. This was especially apparent, reflected in recurring requests for breaks, reduced smiling, limited verbal engagement, and brief engagement responses such as *“I don’t know”*. Environmental distractions, such as nearby slides or playground equipment, also had a more pronounced impact on younger children’s attention toward the end of the session. To maintain engagement, it became necessary to make compromises, offering a few minutes of free play before resuming the discussion.

When asked to rate the difficulty of the tasks, most younger children described them as easy and expressed confidence in their ability to perform them. In contrast, older children were more likely to express doubt or hesitation, often stating, *“I can’t do it”*. Most were unable to explain the reason, responding simply: *“I don’t know, I just can’t” (A 10-year-old girl with Ehlers-Danlos Syndrome)*. However, some children offered explanations linked to their individual abilities: *“Well, I can’t do that! I can’t swing my arm and [lift the leg] at the same time because otherwise I’ll lose my balance” (A 6-year-old girl with CP, using a walker)*. A participant also noted that the difficulty of some skills varied depending on specific technical aspects of the movement. Regarding the skill “striking a moving ball”, she explained: *“It is easy if you hit [the ball] in the right spot, otherwise it is hard” (A 10-year-old girl with fine motor difficulties)*. More specifically, in her justification, she referred to the bounce of the ball, emphasizing that when the ball rebounded close to her, the skills became easier to perform, whereas if the ball bounced too far away, striking it became much more difficult.

### Theme 2: Adaptation Strategies Influence FMS Execution

Adaptation strategies used to support FMS performance were categorized into four subthemes: motor, equipment, environmental, and contextual strategies. These adaptations generally varied based on individual characteristics rather than specific skills. It is important to note that most strategies were initially proposed by the research team and were adjusted with the participants, as children often responded *“I don’t know”* when asked what might help them perform the skill.

#### Motor Strategies

Several motor strategies were observed to support skill execution. The most common was modifying the movement pattern, particularly for the skill “striking a moving ball”. Participants often struck the ball above their head instead of at hip level. Some children justified their execution strategy by linking it to physical activity: *“That’s how we play tennis” (A 9-year-old boy with DCD)*. Similarly, other children have noted that this modification makes it easier for them to perform the skill: *“When I do it like this [demonstration], I find it easier because I can see where it [the ball] is going […] that is how I play” (A 10-year-old girl with fine motor difficulties)*.

Other strategies included skipping certain steps, like omitting the initial swing when throwing, to make the movement easier, or reducing motor demands, especially for younger children. For instance, the tasks of throwing a ball could be simplified by asking them to roll it along the ground rather than projecting it into the air. Another example involved the skill of hopping. Instead of asking the child to cover a certain distance while hopping on one foot, the task was adapted to have them hop in place on one foot.

#### Equipment Strategies

Adapting the equipment was a commonly used strategy, mainly by adjusting object properties (e.g., size, weight, hardness, and colour). These adjustments were made collaboratively with the children to align with their preferences.

For instance, the size of the ball appeared to facilitate catching. Two 3-year-old children, respectively with congenital hypoplasia and agenesis with adactyly (resulting in the absence of left-hand), found it easier to catch larger balls. This preference was also seen more generally among younger participants. In contrast, for throwing skills, all children preferred smaller balls, which were easier to grasp with one hand. Larger balls typically require two hands. Anticipating this, a participant remarked, *“It is going to be harder, I think” (A 6-year-old girl with cerebral palsy)*, and showed hesitation when holding the ball, unsure of her grip. Similarly, size preferences extended beyond balls. A participant mentioned after failing to strike the ball: *“If I had a bigger racket, it would have struck [the ball]” (A 9-year-old boy with DCD*).

Weight was also a factor. Most children favoured lighter balls, although they rarely explained why. Some preferred heavier balls for striking or kicking tasks, but again, no clear reasons were provided. In terms of hardness, many participants chose soft balls (e.g., foam) because they were easier to grasp. Some individuals expressed a preference for firmer balls (such as plastic), but they did not elaborate on their rationale for this choice. The aspect of the ball’s ability to bounce also influenced preferences: *“I like it [the ball] because it is pretty, but I don’t like it because it doesn’t bounce” (6-year-old girl with cerebral palsy)*. Beyond functionality, some of the younger children were also attracted to balls with bright colours or familiar designs, particularly those depicting popular cartoon characters or patterns.

In some cases, technical aids were needed to support performance. This was observed in a 6-year-old girl with CP who used a walker. While the walker provided balance support, its unstable casters (without brakes) could also cause instability. Moreover, some skills need to use both hands. As a result, she practised object control skills, like catching and overhead throwing while seated, simulating the use of a wheelchair. This adaptation was suggested after she shared that she sometimes used a wheelchair. During catching skill, she stated: *“It is easier when I’m sitting on a chair” (A 6-year-old girl with CP)*, though she often let the ball land on her knees rather than using her hands and needed verbal prompts to engage her upper limbs. She did not express a clear preference between using the walker and simulating the wheelchair. For overhead throwing, she voiced concerns before the attempt: *“The difference is that when I’m in my wheelchair and I have a ball and I want to throw it, it doesn’t go far […] I find it better with my walker” (A 6-year-old girl with CP)*. After trying both, she indicated she liked both options but preferred the walker due to familiarity.

#### Environmental Strategies

One commonly used approach was adjusting the distance between the child and the evaluator or target. For example, in younger children, the standard TGMD-3 distance was often shortened to improve their chances of catching the ball or successfully reaching the wall during the underhand throw (criteria 3).

Another key strategy was the use of physical support. For children experiencing balance challenges, assistance such as holding a hand or offering a stable point of contact helped increase their sense of security. This allowed them to concentrate more fully on the task without fear or falling. Physical support also encouraged hesitant children to try skills they were initially reluctant to preform, particularly during more demanding skills like hopping and sliding.

Additionally, in cases where assistive devices were used, such as walkers, additional human support was sometimes needed to stabilize the equipment. This was observed in a 6-year-old girl with cerebral palsy, where the walker’s design (casters without brakes) created instability during overhead and underhand throwing tasks. Holding the walker in place increased safety and focus on the task.

#### Contextual Strategies

Contextual strategies differed by age group. For younger children (ages 3–5), sessions integrated playful elements. Activities were framed as games; for example, children rolled up a die featuring familiar cartoon characters, each associated with a specific FMS and a challenge. In the underhand throw skill, children were asked to knock down pins by rolling a ball along the floor. Similarly, for the gallop skill, they completed laps around the gymnasium using a hobbyhorse, making the movements more accessible and enjoyable.

For older children (ages 6–10), sessions followed a more structured format. However, connecting the skill to a familiar sport or activity appeared to enhance movement execution for some children, especially for object control skills. For example, several participants naturally linked the two-hand strike of a stationary ball to baseball. One 8-year-old child, with DCD and who reported frequent participation in sports, asked to strike a moving ball instead, to simulate an actual game. During this version of the task, he spontaneously performed the movement in full accordance with TGMD-3 performance criteria. He later said he did not realize he had made it “the right way”. The same child also met all the criteria for the dribble skill when it was reframed as dribbling toward a basketball hoop to score.

### Theme 3: Varied Perceptions of Skill Execution

Most children, regardless of age, were unable to identify differences between the demonstration and evaluation videos. Only a few children, mainly those aged 8 to 10, were able to articulate distinctions between the two. For the underhand throw, several noted differences related to the outcome-based success criterion (criterion 3), especially the height of the throw.

Interestingly, some children described differences that were not tied to the TGMD-3 performance criteria. Instead, their observations focused on more general aspects of performance. For example, a participant said: *“Well, I noticed that you [evaluator] jumped farther” (a 10-year-old girl with Ehlers-Danlos Syndrome)*. Another child commented: *“She [evaluator] throws higher than me, for sure […] because she [evaluator] is taller” (A 6-year-old girl with cerebral palsy using a walker)*. In some cases, children also focused on whether the ball hit the wall, even though this was not part of success criteria (e.g., strike a moving ball, kicking, overhead throwing).

## Discussion

This study aimed to explore how children with physical disabilities perceive and perform FMS. Findings reveal considerable variability in their understanding of these skills, influenced by factors such as age, context of evaluation and the use of videos or demonstrations. Motivation and perceived competence also fluctuated, shaped by both individual (e.g., age, fatigue) and environmental (e.g., distractions, session duration) factors. Children used various adaptation strategies (i.e., motor, equipment, environmental, contextual), which supported their engagement and performance. Their perceptions of performance often emphasized subjective elements not captured by standardized assessment, highlighting a gap between lived experiences and conventional evaluation norms.

An element that must be considered when interpreting these findings is the heterogeneity of the children’s diagnoses and associated comorbidities. Children with physical disabilities rarely present with a single, isolated condition; rather they often experience co-occuring motor, sensory, cognitive, attentional or perceptual difficulties that influence how they understand, perform and adapt motor tasks (Blank et al., 2019; Novak et al., 2017). For example, variations in muscle tone, motor planning, visual-perceptual processing, fatigue or attentional regular (common across conditions such as CP or DCD) can shape both the strategies children use and their perceived competence (Blank et al., 2019; Martinie et al., 2021; Novak et al., 2017; Poitras et al., 2021). These comorbidities may therefore contribute to the differences observed across skills and age groups in our study, not as confounding factors, but as integral components of children lived motor experiences. Acknowledging this diagnostic variability is essential for contextualizing our results and reinforces the need for individualized, flexible and participation-focused approaches to motor development.

The results reveal a heterogeneous understanding of FMS among children with physical disabilities. While familiar skills such as running or jumping were generally recognized, others, including galloping or sliding, were less understood. These findings align with Logan et al. (2011), who emphasized that structured teaching, repeated practice and reinforcement are essential for developing FMS (Logan et al., 2011). Without such opportunities, rarely encountered FMS remain underdeveloped. Moreover, the lack of familiarity may also be attributed to context: children appeared to better understand skills they had encountered in familiar or playful environments, reinforcing the theory of situated learning, which views learning as social, contextual and embedded in activity (Lave & Wenger, 1991).

Children also actively used various strategies (i.e., motor, material, environmental, contextual) to adapt their participation in the skills proposed, often in collaboration with the research team. These strategies are aligned with Newell’s constraints model (1986), which views motor performance as emerging from the dynamic interaction between individual, task and environmental constraints (Newell, 1986). These findings highlight the importance of continuing to promote and support personalized motor development approaches that integrate structured, evidence-based methods while remaining sensitive to children’s perspectives and preferences. This is echoed by the recommendations of King et al. (2020), who advocate for recognizing children as active partners in the rehabilitation process. When children help set goals and shape adaptations, their engagement strengthens, motivation increases, and the activities become more meaningful (King et al., 2020). This approach also promotes a therapeutic relationship built on collaboration. Adults (i.e., caregivers, health professionals, teachers) play a crucial role, especially for children who have difficulty expressing their needs (King et al., 2020). In our study, adult input was key, both functionally relevant and consistent with the child’s lived experience, even when preferences were not explicitly voiced.

The findings also suggest that younger children showed strong confidence in their motor ability. This aligns with the notion of developmental optimism, a common tendency, particularly among children under the age of 8, to overestimate their abilities and underestimate the difficulty of task (Harter & Leahy, 2001). This optimistic perspective may encourage children to explore new situations with curiosity and engagement, as they are less constrained by an awareness of their limitations (Lipko et al., 2009). While both boys and girls exhibit this optimism in early childhood, later developmental stages show emerging gender differences in self-evaluations, influenced by social and contextual factors (Harter & Leahy, 2001). Developmental optimism is directly reflected in their perceived motor competence, which is defined as an individual’s subjective evaluation of their ability to perform motor skills (Estevan & Barnett, 2018). It plays a key role in children’s motivation and participation in physical activity (Estevan & Barnett, 2018). However, previous studies have highlighted a gap between perceived and actual motor competence in children with or without disabilities (De Meester et al., 2016; Robinson et al., 2015), but this gap appears to decrease with age and experience (Barnett et al., 2008). In the present study, older children expressed more doubt and self-critical about their abilities, which may indicate growing awareness of their limitations. This likely reflects the increasing influence of social interactions and peer comparisons on self-perception, a well-documented phenomenon among children with physical disabilities for whom social experiences play a key role in shaping self-concept (Nadeau & Tessier, 2011; Renick & Harter, 1989; Shields et al., 2007).

Another notable finding was the difficulty children had in evaluating their own motor performance, particularly when exposed to video demonstrations. Most children struggled to identify discrepancies between their execution and the demonstration performance, confirming well-established limitations in motor self-assessment (Barnett et al., 2016; Robinson et al., 2015). Moreover, visual feedback, especially video modelling, has been shown to support skill acquisition by reinforcing mental representations of movement (Mödinger et al., 2022; Obrusnikova & Rattigan, 2016). Having the possibility to show children their own performance can further enhance self-awareness and promote deeper understanding. This approach can be particularly helpful for children with specific needs, such as attention disorders, motor delays or information processing difficulties. Video prompting, which breaks action sequences into smaller steps, is especially recommended for young children or those with cognitive disabilities, as it reduces cognitive load and facilitates step-by-step learning (Obrusnikova & Rattigan, 2016). Furthermore, videos have been shown to limit distractions, boost motivation and promote autonomy, all of which are critical benefits for children with disabilities (Mödinger et al., 2022; Obrusnikova & Rattigan, 2016). Additionally, the integration of visual and auditory feedback in videos can enhance memory retention, especially in children with verbal and attentional difficulties (Obrusnikova & Rattigan, 2016). However, a previous study offered a nuanced perspective for children aged 8 to 9: immediate and continuous verbal feedback, focusing on movement execution, was more effective for learning some basic motor skills (Mödinger et al., 2022). This suggests that feedback modality should be tailored to the child’s age, cognitive profile and the specific skills targeted (e.g., overhead throwing, catching, horizontal jumping, and dribbling).

Our data also show that children tend to focus more on the outcome of a movement (e.g., hitting a target) than the quality of the movement was performed (e.g., posture, coordination, movement path). This outcome-focused attention reflects the distinction between product- and process-oriented assessment of FMS. Product-oriented assessment may be more intuitive and accessible, especially for children with CP (Capio and Abernethy, 2011). This supports the need to combine both assessment types to better capture motor competence (Hebinck et al., 2025; Logan and Stodden, 2017). The outcome of the movement is potentially more concrete and immediately observable, making it a more accessible reference point for children than the technical aspects of the movement.

This distinction also echoes the difference between Knowledge of Results (KR), which provides outcome-based feedback, and Knowledge of Performance (KP), which targets movement quality feedback (Oppici et al., 2024). While children in our study focused on results, studies show that KP is more effective than KR alone for promoting long-term retention of motor skills, especially when it is prescriptive (i.e., including specific corrective instructions) (Oppici et al., 2024; Robert et al., 2017).

The frequency and timing of feedback also play a critical role in motor learning, particularly in children with physical disabilities such as cerebral palsy. In this population, continuous KR may enhance short-term performance but can hinder long-term retention by fostering dependency on external cues and limiting intrinsic feedback use (Hemayattalab & Rostami, 2010). In contrast, faded feedback (i.e., gradually reduced over time) fosters greater motor autonomy and deeper cognitive engagement, encouraging children to reflect and develop internal error-detection strategies (Hemayattalab & Rostami, 2010). Similarly, well-time delayed feedback allows children to process their own sensory experience before receiving external input, reinforcing internal movement representations (Oppici et al., 2024). For children with DCD, KR may be useful for simple tasks, but more complex motor skills generally require the complementary use of KP, delivered with reduced frequency and adjusted time (Welsby et al., 2024). Overall, a strategic combination of KR and KP, progressively adapted in timing and intensity, appears most effective in supporting both learning and retention, especially in children with atypical development (Oppici et al., 2024). Since children naturally attend to results, it is essential to help them engage with the process, not by opposing these two perspectives, but by integrating them into complementary components of motor development.

Although this study focuses on how children with physical disabilities perceive and perform their FMS, it is important to recognize that this perspective is primarily based on standardized assessments, which may reflect an ableist approach (Giese et al., 2023). Norm-referenced tools such as the TGMD-3 have been criticized for emphasizing deficits by comparing children’s performance to a presumed standard of normality, thereby reinforcing ableist norms and potentially devaluing diverse ways of moving (Giese et al., 2023). Research also shows that therapists frequently adapt these assessments to meet the needs of children, highlighting a disconnect between rigid testing protocols and the realities of clinical and educational contexts (Fay et al., 2018). The strategies identified in our study may help professionals in tailoring assessments and activities to better support diverse learners. While FMS can be valuable in promoting participation in physical activity, this focus should not eclipse other meaningful forms of engagement and competence for children with disabilities. A shift in perspective toward the quality of participation, rather than solely the quality of movement, would foster a more inclusive approach, one that values autonomy, motivation, social interaction, and contextual relevance over technical performance (Giese et al., 2023). Future research should explore ways to involve children directly in the co-construction of assessment tools, empowering them by placing their lived experiences and knowledge at the centre of decisions that affect them (Giese et al., 2023; Giese et al., 2023).

### Limitations

This study presents few limitations that should be acknowledged. All sessions were conducted, recorded, assessed and analyzed by the same person. While a reflexive journal was maintained to mitigate subjective bias, the dual role of facilitator and evaluator may have influenced the interpretation of the data, particularly through implicit expectations or expert assumptions (Lincoln & Guba, 1985). Moreover, the study was conducted entirely within a single controlled environment. While this provides consistency across sessions, it limits the ecological validity of the results (Bonney & Smits-Engelsman, 2019; Bronfenbrenner, 1977). The setting does not fully reflect the real-world contexts in which children typically engage in motor activities, such as schools or at home. The unfamiliar setting and the presence of an unknown adult may have children’s comfort levels and verbal expressiveness during the sessions, potentially limiting the richness of the collected data (Clark, 2005; Hill, 2006). Furthermore, although the presence of parents provided valuable support to their child when needed, it may also have inadvertently shaped the children’s behaviors or responses, thereby adding variability to the data collection process (Einarsdóttir, 2007; Hill, 2006; Irwin & Johnson, 2005).

## Conclusion

This study highlights the diversity of experiences and strategies used by children with physical disabilities in performing FMS. The findings emphasize the importance of contextualized instruction, individualized adaptations, and feedback tailored to each child’s age and motor profile. Recognizing children as active participants and combining both process- and product-oriented approaches are key to enhancing engagement, motivation, and long-term motor learning. These insights call for flexible and inclusive rehabilitation and educational practices that are grounded in children’s lived experiences. Future research should explore how these strategies can be systematically integrated across various settings, including in children’s everyday environments.

## Supplemental Material

Supplemental Material - Perceptions and Adaptation Strategies in Fundamental Movement Skills Among Children With Physical Disabilities Supplemental Material for Trust in Perceptions and Adaptation Strategies in Fundamental Movement Skills Among Children With Physical Disabilities by Margaux Hebinck, Krista L. Best, Maxime T. Robert in Perceptual and Motor Skills.

Supplemental Material - Perceptions and Adaptation Strategies in Fundamental Movement Skills Among Children With Physical Disabilities Supplemental Material for Trust in Perceptions and Adaptation Strategies in Fundamental Movement Skills Among Children With Physical Disabilities by Margaux Hebinck, Krista L. Best, Maxime T. Robert in Perceptual and Motor Skills.

Supplemental Material - Perceptions and Adaptation Strategies in Fundamental Movement Skills Among Children With Physical Disabilities Supplemental Material for Trust in Perceptions and Adaptation Strategies in Fundamental Movement Skills Among Children With Physical Disabilities by Margaux Hebinck, Krista L. Best, Maxime T. Robert in Perceptual and Motor Skills.

## Data Availability

The data generated during and/or analyzed during the current study are available from the corresponding author on reasonable request.*
