# Supplementary material for: Perceptions and Adaptation Strategies in Fundamental Movement Skills Among Children With Physical Disabilities
Source: Percept Mot Skills. 2025 Dec 24;133(6):1905–30. doi: 10.1177/00315125251412691 (PMC13724812; doi:10.1177/00315125251412691)
Supplement: Supplementary file 2 [file sj-pdf-2-pms-10.1177_00315125251412691.pdf]

Skills	Proposed adaptations based on the literature
**Locomotor skills**	
Gallop	Task decomposition (older children); use of a stick horse (younger children)
Hop	Progressive instruction: e.g., hop in place inside a hoop → hop forward out of the hoop → attempt multiple hops in a row
Skip	Task decomposition (older children); use of floor markings
Horizontal jump	Use of floor markers to encourage children to challenge themselves and jump further than the previous attempt
Slide	Use of a mirror
**Object control skills**	
Two-hand strike	Variation in type or size of the ball
One-hand strike	Variation in type or size of the ball
Dribble	Variation in type or size of the ball
Catch	Variation in type or size of the ball
Kick	—
Overhand throw	Variation in type or size of the ball
Underhand throw	Variation in type or size of the ball

Supplementary Material 2. Proposed adaptations based on the literature for locomotor and object control skills (Colvin et al., 2023; Department of Education Western Australia, 2013)**.** For the "kick" skill, no evidence-based adaptive strategies were used, as this skill was discussed only with the child with a transtibial amputation. In this case, the focus was placed on how this child spontaneously performed the movement, and the discussion centered primarily on her own way of executing the task.
