# Supplementary material for: Perceptions and Adaptation Strategies in Fundamental Movement Skills Among Children With Physical Disabilities
Source: Percept Mot Skills. 2025 Dec 24;133(6):1905–30. doi: 10.1177/00315125251412691 (PMC13724812; doi:10.1177/00315125251412691)
Supplement: Supplementary file 3 [file sj-pdf-3-pms-10.1177_00315125251412691.pdf]

Skill	Number of participants who recognised the skill by name		
	3-5 years *(N/Total (%))*	***6-7 years*** *(N/Total (%))*	***8-10 years*** *(N/Total (%))*
**Locomotion skills**			
Gallop	0/2 (0%)	-	1/3 (33%)
Hop	4/4 (100%)	2/2 (100%)	3/3 (100%)
Skip	0/1 (0%)	0/4 (0%)	0/4 (0%)
Horizontal jump	1/1 (100%)	3/3 (100%)	1/1 (100%)
Slide	0/1 (0%)	0/1 (0%)	1/1 (100%)
**Object control skills**			
Two-hand strike	1/1 (100%)	-	2/4 (50%)
One-hand strike	0/1 (0%)	0/1 (0%)	3/5 (60%)
Dribble	4/4 (100%)	1/1 (100%)	3/3 (100%)
Catch	2/2 (100%)	2/2 (100%)	1/1 (100%)
Kick	-	-	1/1 (100%)
Overhand throw	0/1 (0%)	2/3 (67%)	2/3 (67%)
Underhand throw	0/1 (0%)	1/2 (50%)	1/2 (50%)

Supplementary Material 3. Number of participants who recognised each fundamental movement skill by name, stratified by age group.
